# An Agenda for Public Health Diplomacy in an Age of Populism

**DOI:** 10.3389/phrs.2025.1609089

**Published:** 2025-12-05

**Authors:** Martin McKee, Josep Figueras, Ramune Kalediene, Ashish Joshi, Ayman El-Mohandes, Robert Otok, Laura Magaña, Charlotte Marchandise, Henrique Barros

**Affiliations:** 1 European Public Health Association (EUPHA), Utrecht, Netherlands; 2 London School of Hygiene & Tropical Medicine, London, United Kingdom; 3 Association of Schools of Public Health in the European Region (ASPHER), Brussels, Belgium; 4 Lietuvos sveikatos mokslu universitetas, Kaunas, Lithuania; 5 Association of Schools and Programs of Public Health, Washington, DC, United States; 6 School of Public Health, University of Memphis, Memphis, TN, United States; 7 The City University of New York (CUNY) Graduate School of Public Health & Health Policy, New York, NY, United States; 8 Universidade do Porto Instituto de Saude Publica, Porto, Portugal

**Keywords:** diplomacy, public health, politics, human rights, advocacy

## Abstract

**Background:**

Public health is under pressure from rising populism, disinformation, and weakened global institutions, threatening cooperation, equity, and trust in science.

**Analysis:**

Populism politicises health, suppresses evidence, and marginalises vulnerable groups. Public health diplomacy must adapt, becoming more politically aware, ethically grounded, and resilient.

**Policy Options:**

We propose nine ways forward: create diplomacy labs for crisis simulation; empower non-state actors like cities and NGOs; strengthen ethical communication and listening; protect health workers; build alternative accountability systems; reframe health as a diplomatic priority; decentralise and diversify funding; develop a new diplomacy curriculum; reinvent and defend multilateralism.

**Conclusion:**

Public health diplomacy must evolve into a bold, inclusive, and strategic force. By defending evidence, empowering diverse actors, and reforming institutions, it can safeguard health as a foundation for peace and global progress.

## Background

Public health stands at a critical crossroads. In recent years, the global political landscape has been reshaped by the rise of populism, the spread of disinformation, and the weakening of multilateral institutions. These forces have eroded trust in science, undermined human rights, and disrupted international cooperation, core pillars upon which public health progress depends.

This is especially shocking given how much public health, working across borders, has achieved. From the early efforts of the League of Nations’ Health Committee [1], to the eradication of smallpox [2], through WHO-led campaigns, and more recently, to global initiatives that have promoted vaccine equity [3] and strengthened disease surveillance [4], diplomacy has played a vital role in advancing health equity and resilience [5]. It has also enabled humanitarian coordination [6] during crises and fostered capacity-building among health professionals and diplomats [7].

However, the global context has shifted dramatically. The traditional mechanisms of global health governance are increasingly strained. The United States is withdrawing from the WHO, UNESCO and the Paris agreement, undermining multilateral health and climate cooperation with major public health consequences. Structures in other sectors with profound influence over health are struggling, such as the World Trade Organisation, which works on issues such as tariffs on medicines, and the international courts, which play a crucial role in upholding human rights. Attacks on science are reducing the space for evidence-based, cooperative action.

## Analysis

Many of these problems reflect the rise of populist politics. These movements, often characterised by nationalism, anti-intellectualism, and authoritarian tendencies, have disrupted the global health landscape. Once in power, authoritarian populist governments frequently undermine scientific evidence, marginalise vulnerable populations, and politicise health systems, eroding the foundations of transparency, equity, and cooperation [8]. They have politicised previously largely uncontentious health issues, targeted vulnerable populations, and suppressed data, threatening the integrity and effectiveness of public health responses. This situation presents a profound challenge to the principles and practices of public health.

Public health diplomacy offers one tool to confront these challenges. It is the collaborative processes through which governments, international organisations, and non-state actors negotiate and coordinate actions to address global health challenges [9]. It integrates foreign policy, development, and health, drawing on a diverse set of disciplines and skills, including communication, negotiation, systems thinking, and equity-focused approaches, to improve health and wellbeing across borders.

In this new climate, we in the public health community, who have long depended on an acceptance of the importance of scientific expertise and multilateral collaboration, must now contend with a more adversarial and fragmented environment. The erosion of trust in institutions and the proliferation of misinformation have created fertile ground for narratives that distort public health goals. Consequently, it must evolve beyond its conventional boundaries to become more politically literate, ethically grounded, and strategically agile.

To respond effectively, we must embrace a more inclusive and resilient model, one that decentralises authority, empowers civil society, and defends the integrity of evidence and ethics.

While our agenda emphasises the importance of empowering subnational and non-state actors, particularly when national governments are compromised, we also recognise that the state remains a vital actor. Bureaucracies often retain professional norms and institutional memory that can moderate or resist populist agendas. Moreover, non-state actors frequently rely on alliances with foreign governments to exert pressure on domestic regimes. Our approach therefore advocates for flexible engagement: leveraging state institutions where possible, while building complementary networks of influence through civil society, academia, and local authorities.

## Policy Options

We propose nine ways to revitalise and future-proof public health diplomacy. These are designed to move beyond traditional, state-centric approaches and embrace a multilevel, resilient, and ethically grounded model of global health engagement. Each addresses a specific vulnerability in the current system and offers a practical pathway to strengthen capacity to operate effectively in adversarial political environments. We indicate who the target audience is for each one. Together, they form a bold and adaptive agenda for safeguarding global health cooperation and equity in an age of populism.

The first, for public health schools, ministries, and international organisations is to establish mechanisms for developing and rehearsing responses to emerging threats. We must be proactive, not reactive. Virtual laboratories offer spaces for simulation, negotiation, and co-creation and enable rehearsal of responses to crises before they unfold. Public health diplomacy labs would function as interdisciplinary, simulation-based environments where diplomats, public health professionals, civil society actors, and others collaboratively rehearse responses to emerging global health threats. These labs would combine scenario planning, negotiation exercises, and systems thinking to build capacity for navigating complex political landscapes. Drawing inspiration from emergency preparedness simulations used by WHO and national health agencies [10], as well as policy innovation labs like the OECD’s Observatory of Public Sector Innovation [11], diplomacy labs would foster real-time learning, consensus-building, and strategic foresight. They could also learn from humanitarian diplomacy training platforms (e.g., those used by the Red Cross and ASEAN) and from public health diplomacy summits, such as the University of Memphis model [9]. By integrating experiential learning with political literacy and ethical negotiation, these labs would prepare actors to defend science in increasingly adversarial and populist contexts. They would be incubators for innovation, fostering cross-sector collaboration and building the skills needed to navigate complex political landscapes. By practising diplomacy under pressure, we can prepare for the unexpected and ensure that public health remains resilient in the face of populist disruption.

The second, for cities, regional authorities, and NGOs, is to empower actors other than nation-states. When national governments are compromised by populist agendas, subnational and non-state actors must step forward. Cities, regions, universities, and NGOs often retain the capacity and credibility to challenge populist authoritarian regimes. Moreover, while subnational governments often prioritise local concerns, many have shown leadership in international cooperation, particularly in areas such as climate change and public health. European examples include the resistance by mayors to Viktor Orban’s government in Hungary [12] and the policies implemented by Ekrem İmamoğlu, mayor of Istanbul, in the face of illiberal measures adopted by Turkish President Recep Tayyip Erdoğan [13]. These actors should be supported to form cross-border coalitions, share resources, and advocate for evidence-based policies. While profit-oriented companies may face political and economic constraints, our focus is on civil society, academia, and local authorities, which often retain greater flexibility to uphold public health values. For example, in response to federal decisions affecting the US Centers for Disease Control, state authorities in California, Oregon, and Washington have come together to create a West Coast alliance to align vaccine assessments and decisions [14]. Empowering them means investing in their diplomatic capabilities and creating platforms for coordination. Students and professors in Serbian universities have provided several inspirational examples of how to internationalise their campaigns, using imaginative means to take their message to other European capitals [15].

The third, for public health institutions and media professionals, is to strengthen public engagement [16]. In an age of misinformation, they must engage with the concerns of the population. Public health institutions must listen actively, increasingly aided by digital monitoring of public sentiment to identify emerging concerns and counter disinformation early [17]. They need spokespeople who communicate clearly, credibly, and compassionately, understanding the anxieties, hopes, and expectations of the public [18]. Strategic messaging should be proactive, not reactive, anticipating populist narratives and offering compelling alternatives. Skills in analysing public sentiment when faced with an infodemic are as important as the epidemiological expertise needed to respond to a pandemic [19]. Constant vigilance is required to avoid distortion and censorship, exemplified by the Trump administration’s war on the language of diversity [20]. By institutionalising these practices, public health can reclaim the narrative and build trust with communities that feel abandoned or misled [21].

The fourth, for legal advocacy groups, professional associations, and international bodies, is to protect the health workers and journalists who are often the first targets of authoritarian populist governments. Increasingly, they are being targeted explicitly in conflict zones, with far too many losing their lives [22]. Responses must include mechanisms to protect these individuals from retaliation. Legal defence funds, diplomatic support networks, and international solidarity campaigns should be established to shield those who speak truth to power. Their safety is not just a human rights issue; it is essential to maintaining transparency and accountability in health systems, for example, when addressing corruption, a major but often ignored cause of health system failure [23], and the topic of a recently launched Lancet Commission [24]. By standing together, the public health community affirms its commitment to justice and ensures that critical voices are not silenced.

The fifth, for civil society watchdogs, investigative media, and research centres, is to build alternative accountability systems. One of public health’s most important tasks is to make the invisible, whether people, places, or issues, visible. When official channels are compromised, alternative systems must emerge to uphold transparency. Independent data platforms, verification mechanisms, and civil society watchdogs can provide credible information and monitor abuses. These organisations should support these efforts by partnering with investigative media and advocacy organisations [25]. Strategic litigation is also a core part of the public health armamentarium [26], and legal action is one of the very few tools that has been effective in preventing some of the most egregious actions of the Trump administration [27]. These systems must be agile, secure, and trusted, capable of operating in hostile environments and reaching global audiences.

The sixth, for foreign ministries, health departments, and policy think tanks, is to reframe public health as a diplomatic priority. Health is not just a technical concern; it is a strategic asset. These organisations must position health as central to foreign policy, national security, and economic resilience. This is a shift from Health *in* all Policies to Health *for* all Policies [28]. This means advocating for assessing the impact of health in all major policy domains, from climate and migration to trade and technology. Public health leaders must engage with diplomats, economists, and security experts to make the case that health is foundational to stability and prosperity. By reframing health as a diplomatic priority, we elevate its importance and embed it in the core of global decision-making.

The seventh is to diversify and decentralise funding. Populists often politicise funding, threatening the independence of public health institutions [29, 30]. To counter this, donors, foundations, and multilateral organisations must pursue diversified and decentralised financing. Multilateral organisations, philanthropic foundations, and regional networks should be mobilised to provide flexible, reliable support. Funding mechanisms must be designed to respond rapidly to emerging threats and to sustain long-term capacity building. By reducing dependence on politicised national budgets, we safeguard the autonomy of public health actors and ensure continuity in times of political instability.

The eighth, for academic institutions and training bodies, is to prepare future leaders with political literacy and negotiation skills. The next-generation of public health leaders must be equipped not only with technical expertise but with diplomatic acumen. A new curriculum is needed, one that integrates ethics, negotiation, political literacy, systems thinking, and strategic communication. Training should be experiential, interdisciplinary, and globally oriented. Public health education must prepare students to navigate complex political environments, engage with diverse stakeholders, and advocate for equity and evidence. By investing in this curriculum, we build a cadre of leaders ready to defend public health in an age of populism.

The final task, for international organisations, national governments, and civil society coalitions, is to defend and reinvent multilateralism. Multilateral institutions are under attack and should be defended. This does not exclude change, but reforms should be pragmatic, avoiding wholesale reinvention. Public health voices must be present in treaty negotiations, standard-setting processes, and international forums. We must advocate for stronger global norms and mechanisms that protect health from political interference. Reinventing multilateralism means building alliances across sectors and regions, ensuring that public health remains a unifying force in a divided world.

Civil society organisations, such as those represented by the authors of this paper, have a critical role to play in advancing the new agenda for public health diplomacy. In an era where populist agendas may compromise national governments, they often retain the credibility, agility, and grassroots connections needed to advocate for evidence-based health policies. The proposed agenda calls for empowering these actors through enhanced diplomatic training, strategic partnerships, and access to decentralised funding streams. It also calls for the removal of barriers to international collaboration, such as visa bans, blocks on financial transfers, and restrictions on electronic exchanges. By providing mutual support, especially valued by public health organisations facing persecution, such groups can help counter misinformation and rebuild trust. Their involvement is not peripheral; it is central to ensuring that organisations contributing to public health remain resilient, inclusive, and responsive in politically hostile environments.

## Conclusion

This is a call to reimagine public health diplomacy, not as a technical exercise, but as a moral and strategic imperative. In an era defined by populism, disinformation, and political obstruction, the stakes for global health have never been higher. The traditional tools of diplomacy, while valuable, are no longer sufficient on their own. We must act boldly, speak truth to power, and build alliances that protect the vulnerable and uphold the values of science, equity, and justice.

The agenda outlined in this paper offers a roadmap for a more courageous, inclusive, and adaptive approach. While the reach of public health diplomacy may be uneven, especially in regions affected by populist politics, our agenda is designed to build resilience through diverse actors and strategies, including inclusive communication and local innovation. It recognises that resilience in the face of political hostility requires not only institutional reform but also a cultural shift, toward ethical leadership, decentralised action, and strategic foresight. By empowering non-state actors, defending truth-tellers, and reinventing multilateralism, we can ensure that public health remains a force for unity and progress.

Public health diplomacy must become a beacon of hope in a fractured world. It must defend the integrity of evidence, amplify the voices of the marginalised, and foster cooperation across borders and sectors. In doing so, it can help build a more just and sustainable future, one where health is not a casualty of politics, but a cornerstone of peace and prosperity.
